# Nano-biochar–nano-calcium oxide synergies enhance yellow-brown soil health and tea productivity via microbial, enzymatic, and genetic pathways

**DOI:** 10.3389/fmicb.2026.1788651

**Published:** 2026-03-09

**Authors:** Sadam Hussain, Chunmei Gong, Usman Zulfiqar, Mayank Anand Gururani, Abdulrahman Alasmari, Nazih Y. Rebouh

**Affiliations:** 1 College of Horticulture, Northwest A&F University, Yangling, China; 2 Department of Agronomy, Faculty of Agriculture and Environment, The Islamia University of Bahawalpur, Bahawalpur, Pakistan; 3 Department of Biology, Nakhchivan State University, Nakhchivan, Azerbaijan; 4 Department of Biology, College of Science, United Arab Emirates University, Al Ain, United Arab Emirates; 5 Department of Biology, Faculty of Science, University of Tabuk, Tabuk, Saudi Arabia; 6 Biodiversity Genomics Unit, Faculty of Science, University of Tabuk, Tabuk, Saudi Arabia; 7 Institute of Environmental Engineering, RUDN University, Moscow, Russia

**Keywords:** drought, genetic pathways, nano-biochar, soil microbes, soil–plant interactions

## Abstract

Tea (*Camellia sinensis* L. O. Kuntze) is an important economic crop widely cultivated in tropical and sub-tropical regions, where drought stress often limits its growth and productivity. Soil application of nano-biochar (nBC) and nano-calcium oxide (nCaO) offers a promising approach for enhancing soil health, tea quality, and yield. A pot experiment was executed to explore the synergistic effects of nBC and nCaO on soil enzymatic and microbial activities, N-P-C cycling genes, and the quality and yield of tea seedlings under drought stress. The results showed that, under drought stress, the combined application of nBC and nCaO significantly improved the soil physico-chemical and microbial properties viz. an increase in soil pH (23.29%), soil organic matter (53.18%), soil total carbon (30.56%), available N (63.12%), available P (140.85%), available K (32.92%), microbial biomass carbon (9.90%) and microbial biomass N (8.23%) compared with the control. This may have been due to manifold increase in the expression levels of N-C-P cycling genes such as *phoD* (5.2-fold), *phoC* (7.0-fold), *narG* (3.4-fold) and *GH31* (1.8-fold) and relatively higher abundance of archaeal and bacterial communities. Soil urease, acid-phosphatase, nitrate reductase, β-glucosidase, catalase, phosphomonoesterase, and *N*-acetyl-β-d-glucosaminidase enzyme values were 48.32, 13.34, 100.00, 43.37, 612.5, 61.30, and 43.65% higher, respectively, in soils amended with both nBC and nCaO than in the control under drought stress. Furthermore, co-application of nBC and nCaO significantly enhanced tea quality traits such as caffeine (5.89%), polyphenol (12.24%), total catechins (11.00%) and amino acid (16.17%), as well as yield parameters including plant height (10.43%), leaf area (97.55%) and 10-bud weight (42.53%) relative to the control. Overall, the combined application of nBC and nCaO substantially improved soil enzymatic and microbial activities, as well as tea quality and yield traits, under drought stress.

## Graphical abstract

**Figure:**
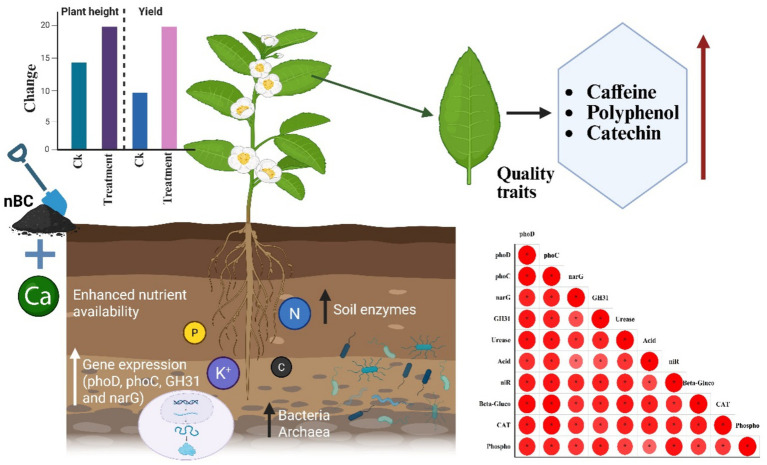

## 1 Introduction

Tea (*Camellia sinensis* L. O. Kuntze), an important perennial woody plant, has unique nutritional value and economic importance worldwide (Shi et al., 2024; Wang et al., 2024; Hussain et al., 2026). Its leaves are widely used to produce non-alcoholic beverages. The crop is predominantly cultivated in the middle and lower reaches of the Yangtze River; i.e., in the regions that are increasingly prone to drought episodes, particularly during the summer season (Cheruiyot et al., 2009; Guo et al., 2017). Drought is one of the major environmental constraints limiting the growth, quality and overall yield of tea (Guo et al., 2017; Qian et al., 2018). Water deficit conditions can result in different disorders at morphological, physio-biochemical and molecular levels in tea seedlings (Rahimi et al., 2019). Some studies have also reported that drought stress retarded tea growth, leaf area, the performance of photosynthetic machinery, reduced pigment formation, and dry matter accumulation (Guo et al., 2008; Hasan et al., 2023). Subsequently, these disorders resulted in significant reduction in the quality parameters and the overall yield (Munivenkatappa et al., 2018). Under water stress, like other crop plants, tea seedlings also show differential adjustments such as changes in basic metabolic processes, numerous structural modifications, greater buildup of secondary metabolites and higher expression levels of stress related genes (Rawat et al., 2017). Different published reports have also demonstrated that tea seedlings reduce stomatal conductance and limit the leaf growth in response to drought stress (Qian et al., 2018; Chaeikar et al., 2020). The development of deeper root systems in tea plants in response to water stress has also been well documented previously. At physiological level, in response to water stress, tea plants have shown to elevate photosynthetic and osmotic regulation, enhance the buildup of antioxidative enzymes, and sustain the production of reactive oxygen species (ROS) under stress condition (Shahbaz et al., 2025; Liu et al., 2016). Although tea plants show several adaptations, yet drought stress causes severe reductions in growth, development and productivity; thus necessitating numerous management tactics to reduce its negative consequence on crop performance.

Nutrient management through organic sources, including soil applied vermicompost and biochar, has shown favorable outcomes to improve drought stress tolerance associated with increased rhizospheric water intake and its retention. Biochar is a carbon-rich organic source that helps improve the performance of soil–plant system. It is formed through pyrolysis process under deficit oxygen environments. Its application in enhancing drought stress tolerance has been well documented (Guo et al., 2022; Shar et al., 2025). Previous studies have stated that soil applied biochar increased soil health in terms of physicochemical and biological properties such as pH, nutrient uptake efficacy, and water retention ability (Alghamdi, 2018; Murtaza et al., 2021). Biochar offers numerous unique porous structures that improve soil aeration properties and reduce soil compaction (Blanco-Canqui, 2017). Furthermore, some studies have demonstrated that biochar has porous surface and thus enhance water absorption and its retention in the rhizosphere. Its application also provides significant benefits under water stress conditions. Likewise, several studies have also demonstrated that biochar can act as a binding agent and promote the development of stable soil aggregates which in turn facilitate root growth and overall crop performance (Wang et al., 2017; Sun et al., 2021). Soil applied biochar helps sustain soil pH and enhance nutrient uptake as most of biochars, particularly produced through agricultural wastes, are alkaline in nature. In addition, on biochar surface, negatively charged functional groups improve soil cation-exchange capacity (CEC), assisting holding of positively charged nutrient ions including potassium and magnesium. Together, all these processes facilitate the growth and yield of crops, particularly under environmental stresses. Soil applied biochar has proved to increase photosynthesis, pigment formation, seedling growth and yield, which were positively associated with higher nutrient uptake, better soil microbial activity and improved relative water content (Khan et al., 2021; Cong et al., 2023). Recently, some studies have also demonstrated that nBC has more pronounced effects on below- and above-ground traits of field crops. For example, Shafiq et al. (2023) reported more pronounced effects of nBC on soil fertility and plant growth due to its nano size. Some studies have also reported its positive role in enhancing drought tolerance in different crops including *Triticum aestivum* (Sammar et al., 2023), *Vicia faba* L. (Abd El-Moaty et al., 2024), *Helianthus annuus* L. (Paneque et al., 2016) and *Chenopodium quinoa* Willd (Tourajzadeh et al., 2024). These improvements are largely associated with better seedling growth, higher photosynthesis and antioxidative defense mechanisms. However, relatively less information is available on the influence of nBC on the performance of tea crop.

Nano calcium oxide also offers essential roles in enhancing drought stress tolerance in crop plants. Calcium plays central role in cell structure maintenance, cell membrane stability, and signal regulation (Mazhar et al., 2022). However, external calcium application is essential because of its poor mobility within plant tissues. Exogenous calcium oxide (CaO) has been found to improve soil physico-chemical and biological properties along with its associated role in enhancing plant growth and photosynthetic performance (Azeez et al., 2021). In another study, Badihi et al. (2021) found that nCaO improves seedling growth and physiological characteristics by enhancing nutrient uptake, plant water status, pigment formation, antioxidative enzymatic activities and ROS metabolism. According to Mazhar et al. (2023), nCaO improved morpho-agronomic traits in carom (*Trachyspermum ammi* L.) seedling through better calcium signaling, and activities of enzymes of the ascorbate glutathione cycle under drought stress. The enhanced drought tolerance under nCaO application has been well demonstrated for different crops including *Brassica napus* (Hou et al., 2022), *Triticum aestivum* (Gao et al., 2023), *Oryza sativa* (Riaz et al., 2025; Minghan et al., 2025) and *Zea mays* (Saeed and Hammok, 2025). However, no study has yet explored its effectiveness for enhancing drought tolerance in tea.

Although individual effectiveness of nBC and nCaO for enhancing stress tolerance is well demonstrated in previous studies (Mazhar et al., 2023; Riaz et al., 2025; Shafiq et al., 2023), relatively less effort has been devoted for exploring the effects of combined application of these amendments under abiotic stresses (Bilge et al., 2025). According to our best knowledge, the effects of nBC and nCaO for enhancing drought tolerance in tea seedlings remain unexplored. Thus, the present study was designed to explore the effects of sole and combined nBC and nCaO application on soil physico-chemical and biological properties and tea performance under drought stress. Specifically, the current work focused on how nBC and nCaO influence soil health, nitrogen use efficiency, leaf quality and yield under drought conditions. This study also explored the synergistic interactions between nCaO and nBC in improving drought tolerance, with analyzing the agronomic potential of these amendments as a sustainable alternative to traditional nutrient managements. We hypothesize that the combined application of nCaO and nBC will exert synergistic effects that enhance drought tolerance and agronomic performance more effectively than their individual applications.

## 2 Materials and methods

### 2.1 Characterization of nano-biochar and nano-calcium oxide

In this study, the nCaO was purchased from Zhongke Yannuo (Beijing) New Material Technology Co., Ltd., China. Scanning electron microscopy (SEM) analysis was performed to analyze the nano materials. Firstly, this nBC was diluted in double-distilled water (DDW). Then, this diluted material was exposed to sonication in an ultrasonic bath for 30 min. The uniform dispersion of the particles was confirmed through SEM analysis while studying the morphological features of nBC. This nBC was purchased from Shaanxi Danong Huitai Bio-health Agricultural Technology Co., Ltd., located in Yangling, China. The morphological characteristics as well as the elemental composition of used nBC have been reported in a previous study of our group (Hussan et al., 2025). Next, a water-dispersible colloidal solution of nBC (w/v) was produced following the sonication and centrifugation procedures described by Lateef et al. (2019). Elemental and nutrient concentrations in the suspension were assessed using ICP-OES (Agilent Technologies 5,110-vdv) after wet-acid digestion, following the US EPA 3050b-1996 standards (US EPA 1996).

### 2.2 Experimentation

This glasshouse experiment was conducted under controlled environmental conditions (25/20 °C (day/night), 75 ± 5% relative humidity, a 12 h light/12 h dark photoperiod, and a light intensity of 300 μmol m^−2^ s^−1^) at the College of Horticulture, Northwest A&F University, China. Yellow-brown topsoil (0–25 cm) was collected and used for all experimental pots. Plastic pots with dimensions of 30 × 25 × 20 cm (top diameter, bottom diameter, and height, respectively) were used, each filled with 5 kg of dry, well-sieved soil. The experiment comprised two factors: drought stress (control [Ck] and drought stress) and soil amendments [control, sole nBC (7.0 g kg^−1^ soil), sole nCaO (100 mg kg^−1^ soil) and combined nBC + nCaO]. The amounts of nBC and nCaO were halved for combined treatments. In drought treatments, the water holding capacity was maintained at 30%, while in well-watered pots, it was maintained at 75%. All seedlings were allowed to grow under normal conditions for 90 days before imposing drought. Tap water was used for irrigation in drought-stressed and well-watered pots. Both nBC and nCaO were applied at the time of soil filling of pots. Three replicates were established for each treatment. Healthy and uniform tea seedlings (cv. *Baiye 1*) were transplanted at the time of pot preparation, with one seedling per pot. All pots were arranged in a complete randomized design with a factorial arrangement. The experiment was continued for 6 months, after which soil and plant samples were collected for further analyses.

### 2.3 Data recording

#### 2.3.1 Soil physico-chemical properties

The pH of the initial and the soil after completion the experiment was measured in a wet soil paste using a pH probe. A soil sample of 1 g was subjected to digestion within a fume chamber through successive addition of nitric acid and hydrogen peroxide. The total N content in the soil was calculated based on the Kjeldahl method (Barbano et al., 1990). Furthermore, phosphorus (P) availability was determined spectrophotometrically, as previously quoted by (Richardson and Reddy, 2013). Soil potassium concentration was calculated using a flame photometer tailored with an acetylene burner (Perkin-Elmer model 52). Soil organic carbon was determined using the Walkley-Black chromic acid moist oxidation technique (Bahadori and Tofighi, 2017). In order to calculate the dissolved organic carbon (DOC), the TOC analyzer (Shimadzu TOC-Vcph, Japan) was used. In addition, total carbon (TC) was calculated using the same method using TOC analyzer.

#### 2.3.2 Microbial biomass and enzymatic activities

The chloroform fumigation-extraction procedure, using K_2_SO_4_, was considered to determine soil microbial biomass performance (Bilenky et al., 2024). Soil urease, acid phosphatase (AP), β-1,4-glucosidase (BG), nitrate reductase and *N*-acetyl-β-d-glucosaminidase (NAG) enzyme activities were quantified using the fluorometric method, as outlined by Tabatabai and Bremner (1972), Dick et al. (2000), Mori et al. (2021), Abdelmagid and Tabatabai (1987) and (ISO_20130, 2018), respectively. In summary, 1 g of oven-dry equivalent fresh soil sample was combined with 100 mL of 50 mM sodium acetate buffer (pH 5.5). Later, this soil was agitated for 1 h in a reciprocating shaker at 200 rpm. The suspension was relocated to a bowl, and an aliquot of 200 μL of soil suspension was extracted. The suspension was kept homogenized by a stirrer in the bowl. This aliquot was then pipetted into 16 wells of a black 96-well plate, in which urea for urease enzyme, 50 μL of 200 μM MUF (4-Methylumbelliferone) substrates 4-MUF-β-d glucopyranoside for BG and NAG enzymes and 4-MUF-phosphate for AP were introduced into the first eight wells designated for the soil assay. In the other eight wells, 50 μL of sodium acetate buffer was added, and then supplemented with 50 μL of MUF (10 μM) standard, with four wells allocated for each in the black plates. Control plates were produced by dispensing 200 μL of buffer and 50 μL of substrate into the first four wells, 200 μL of buffer and 50 μL of MUF into the subsequent four wells, and 250 μL of buffer into another four wells, which correspond to substrate background wells, standard wells, and buffer background wells, respectively. The plates were kept in the dark at room temperature (22 °C) for 3 h before using the fluorometer (Synergy HT, Bio-Tek Instruments, Winoosky, VT, United States) to measure the fluorescence. Lastly, after incubation, an auto-dispenser in the fluorometer delivered 20 μL of NaOH (0.5 M) to each well and shook them well to halt the reaction. The fluorescence was read using an excitation of 360 nm and an emission of 460 nm. Soil catalase activity was quantified based on O_2_ evolution after addition of hydrogen peroxide (3%) using a low flow gas meter (GH Zeal, Merton, London, United Kingdom) (Guwy et al., 1999). The catalase values are expressed as nmol O₂ g^−1^ min^−1^. In addition, the quoted protocol of Yasin et al. (2024) was followed to calculate the phosphomonoesterase activity. The values are shown as nmol p-nitrophenol g^−1^ min^−1^ after 1 h incubation at 37 °C. The average geometric values of all these enzymes were used to quantify the soil quality index. Moreover, soil physical property index, soil chemical property index and soil microbial property index were determined based on MDS (minimum dataset), as briefly described by (Li et al., 2020). For MDS assessment, different soil properties were analyzed with principal component analysis (PCA) and correlation analysis.

#### 2.3.3 N use efficiencies and N enzymes

Agronomic and physiological N use efficiency was determined based on leaf yield and N uptake by seedlings as reported by Shi et al. (2024). Fresh leaf samples were utilized to assess leaf enzyme activity at 90 days after treatments imposition. The activity of nitrate reductase (NR) was assessed following the methodology of King et al. (1992). One milliliter of plant extract was combined with 1 mL of 0.02 M KNO_3_, and the test tube was incubated in dark for 1 h. Subsequent to incubation, 0.5 mL of sulphanilamide was introduced, followed immediately by 0.5 mL of N (1-naphthyl). Upon agitation, a pink color emerged as NO_3_ which was reduced to NO_2_ by NR and finally absorbance was measured at 542 nm. Tris–HCl buffer extraction-based method was followed to determine the activity of glutamine synthetase, detail procedure is described by Cruz et al. (2006).

#### 2.3.4 Alfa diversity of archaea and bacteria

Bacterial soil DNA was extracted from samples utilizing the Power Soil™ DNA Isolation Kit (MO BIO Laboratories, Carlsbad, CA, United States) in accordance with the manufacturer’s guidelines. The bacterial universal primer set, 338F/518R, along with 16S-F (AGAGTTTGATCMTGGCTCAG), 16S-R (GCTGCCTCCCGTAGGAGT) (Tian et al., 2015), and Arch958RmodR (5’-YCCGGCGTTGAVTCCAATT-3′), was utilized to amplify archaeal 16S rRNA genes for real-time PCR analysis.

#### 2.3.5 Soil stoichiometry

Soil ecosystem multifunctionality offers a realistic way to explore the essential role of soil biodiversity. In this work, the measured values of soil enzymes, including BG (C-cycling), urease, NR, *N*-acetyl-β-d-glucosaminidase (NAG, N-cycling), and AP (P-cycling) were used to calculate soil ecosystem multifunctionality. The well-established *Z*-score technique was used to characterize each soil enzyme. The detailed procedure was recently described by (Jia et al., 2025). Moreover, microbial C and other nutrient limitations were quantified by determining the vector length and vector angle (Jia et al., 2025), following the Equations 1 and 2:


$$\text{Vector length}=\sqrt{{\left(\mathrm{BG}/\mathrm{AP}\right)}^{2}+{\left(\mathrm{BG}/\mathrm{NAG}\right)}^{2}}$$ (1)

$$\text{Vector angle}=\text{degrees}[\text{atan}2(\frac{\mathrm{BG}}{\mathrm{AP}},\frac{\mathrm{BG}}{\mathrm{NAG}})]$$ (2)

#### 2.3.6 Growth measurements and quality traits

After 90 days of drought treatments, plant height, leaf area and bud weights were determined. Plant height was measured using measuring tape. Leaf area was quantified based on leaf length and leaf width [leaf area = leaf length × width × 0.7 (correction factor)]. Ten buds were considered from two newly developed leaves to take the bud weight. The average weight of these buds was taken. Total catechin, polyphenol and free amino acids were determined using high-performance liquid chromatography technique, according to the national standard GB/T 83132018. The caffeine concentrations in tea leaves were quantified following GB/T 8312–2013.

#### 2.3.7 Genetic analysis

Three soil samples from each pot were collected, amalgamated into a composite sample, and subsequently freeze-dried for 48 h for further experimentation. In accordance with the manufacturer’s recommendations, DNA was extracted from 0.5 g of lyophilized soil with the FastDNA SPIN Kit (MP Biomedicals, Germany). The quality of DNA was assessed using a NanoDrop ND-1000 spectrophotometer (NanoDrop Technologies, Inc.). The quantification of *phoD* and *phoC* genes was conducted using a Roche LightCycler 96, incorporating three technical duplicates for each biological replicate. Sequencing of 2 × 250 bp amplicons, combined in equimolar quantities, was carried out by Shanghai Personal Biotechnology, Ltd. (Shanghai, China) using the NovaSeq 6,000 SP Reagent Kit (500 cycles) on the Illumina NovaSeq platform. Each biological replicate was accompanied by one technical replicate.

#### 2.3.8 Statistical analysis

All recorded data were analyzed using SPSS 22.0 (IBM Corp., Armonk, NY, United States). A two-way analysis of variance technique was employed to assess the effects of drought stress and organic amendments, and their interactions. The individual effects of drought or organic amendments were quantified by employing one-way analysis of variance. Statistical significant differences in soil physico-biochemical properties, microbial and enzyme activities, nitrogen use efficiency, and quality and yield traits were differentiated using least significance test (*p* < 0.05). Graphical presentation was performed using Origin 2021 software. The relationships between vector length and angle were evaluated using linear regression models. Association between gene expression and soil–plant traits was assessed using Pearson correlation coefficient. Vector angles were used to indicate soil microbial metabolic limitation, whereas soil properties and enzyme activities were considered latent variables.

## 3 Results

### 3.1 Soil physico-chemical properties

**Figure 1:**
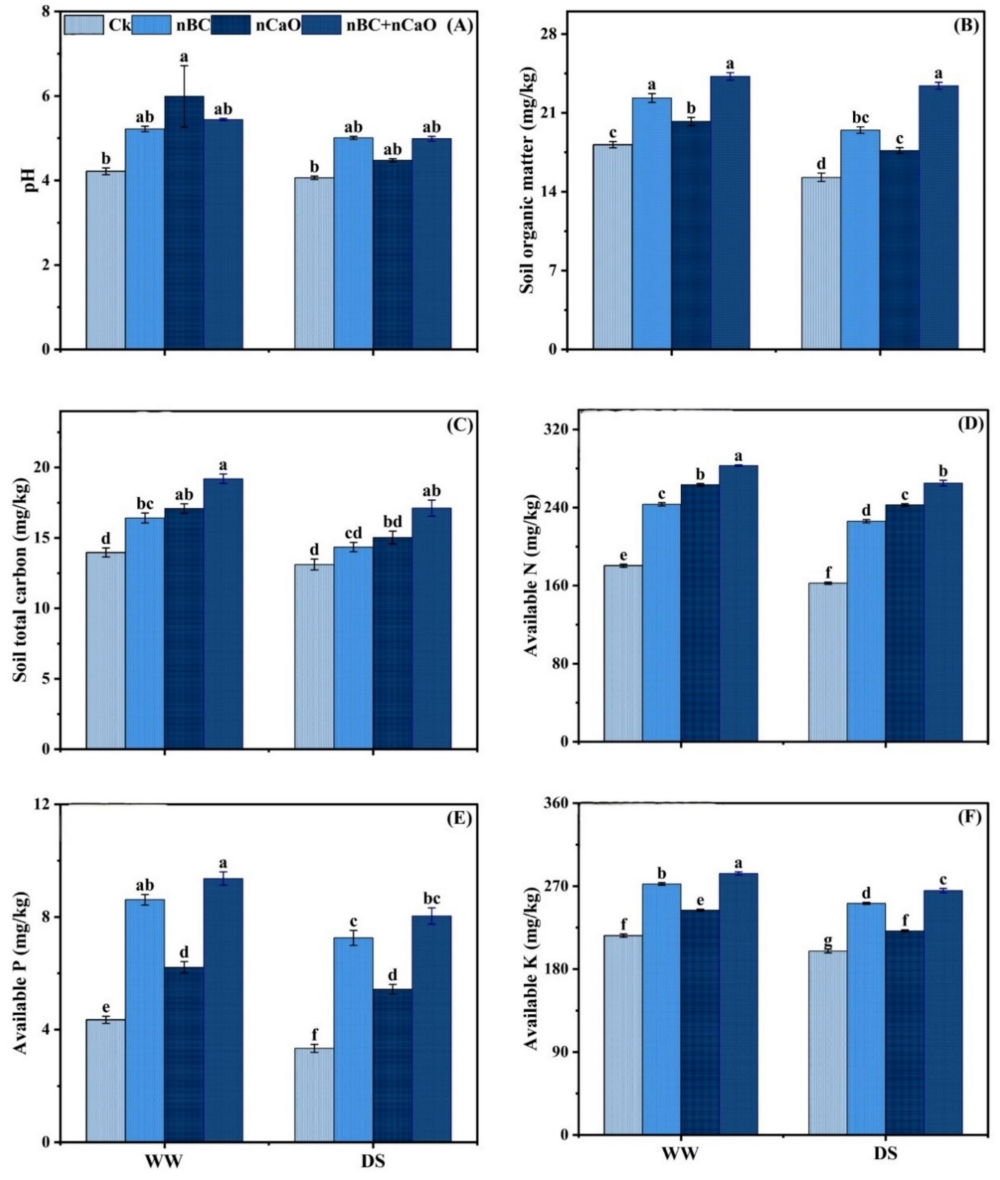
Synergistic impact of nano biochar (nBC) and nano calcium oxide (nCaO) on **(A)** soil pH, **(B)** soil organic matter **(C)** soil total carbon, **(D)** available N, **(E)** available P, and **(F)** available K under well-watered (WW) and drought stress (DS) conditions. The values are mean ± SE of three replicates. Different lowercase letters indicate significant (*p* < 0.05) differences among treatments for WW and DS. Ck, control without nBC and nCaO.

Soil physico-chemical properties varied significantly among drought treatments and nBC and nCaO treatments. Soil pH, organic matter content, total carbon, available N, available P and available K were decreased under drought stress, compared to well-watered conditions (Figure 1). However, these values increased significantly under nBC and nCaO treatments with significantly higher values recorded for combined nBC and nCaO, followed by nBC, nCaO and Ck. Under well-watered and drought stress, co-application of nBC and nCaO significantly increased soil organic matter by 53.18%, soil total carbon by 30.56%, available N by 63.12%, available P by 140.85%, and available K by 32.92%, compared with Ck. Under both well-watered and drought stress conditions, overall soil health performance followed the order: Ck > nCaO > nBC > nBC + nCaO.

**Figure 2:**
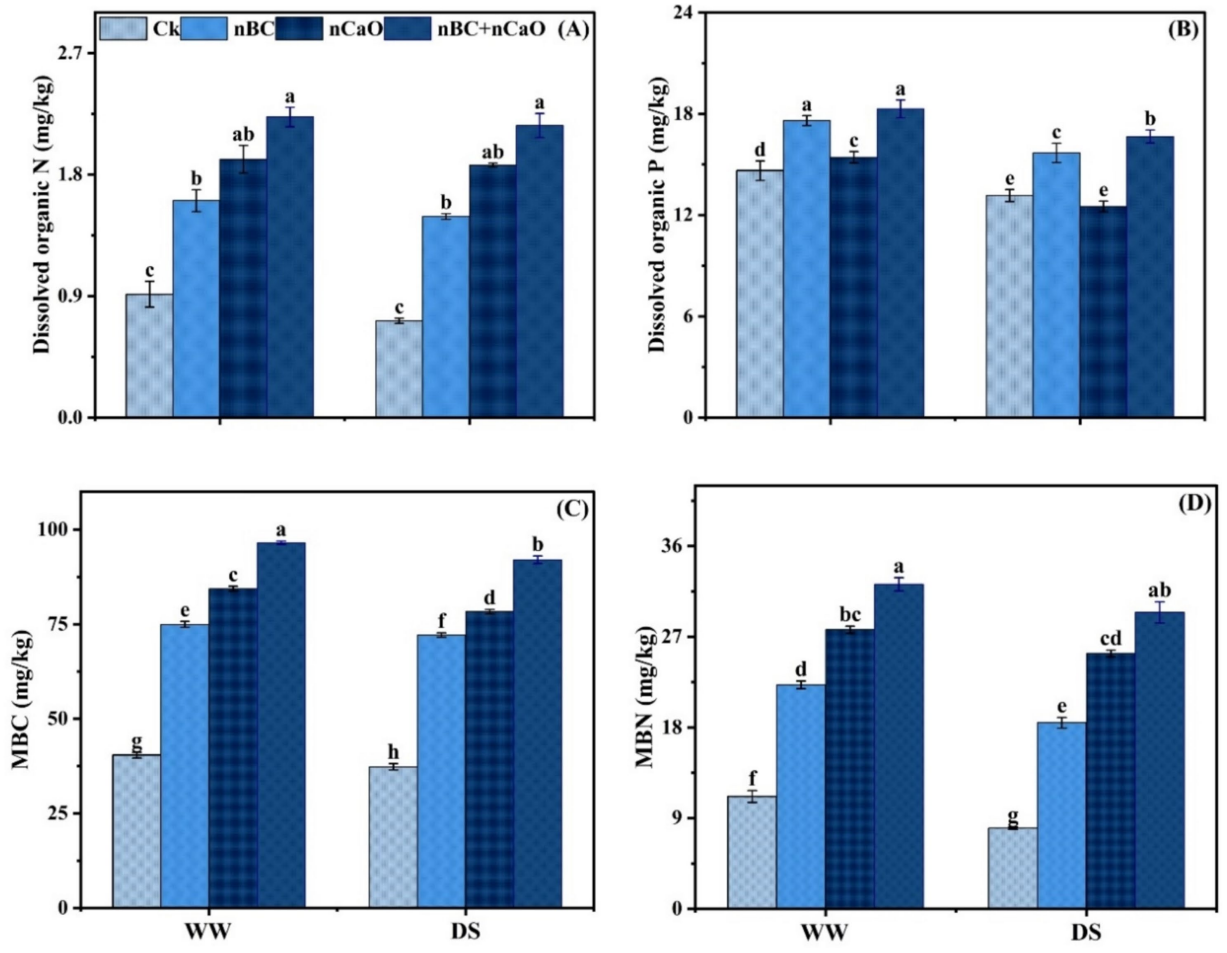
Synergistic impact of nano biochar (nBC) and nano calcium oxide (nCaO) on **(A)** dissolved organic N, **(B)** dissolved organic P, **(C)** microbial biomass carbon (MBC), and **(D)** microbial biomass N (MBN) under well-watered (WW) and drought stress (DS) conditions. The values are mean ± SE of three replicates. Different lowercase letters indicate significant (*p* < 0.05) differences among treatments for WW and DS. Ck, control without nBC and nCaO.

Dissolved organic N, organic P, microbial biomass carbon and microbial N were all influenced by drought stress, which declined the values by 6.02, 11.20, 9.90 and 8.23%, respectively, compared with well-watered conditions (Figure 2). However, nBC and nCaO significantly increased the values of these traits under both well-watered and drought stress conditions. Combined applied nBC and nCaO were more effective, which increased dissolved organic N by 201.80%, organic P by 265.40%, microbial biomass carbon by 146.60% and microbial N by 267.70%, respectively, compared to control, under drought stress conditions (Figure 2).

### 3.2 Soil properties index

**Figure 3:**
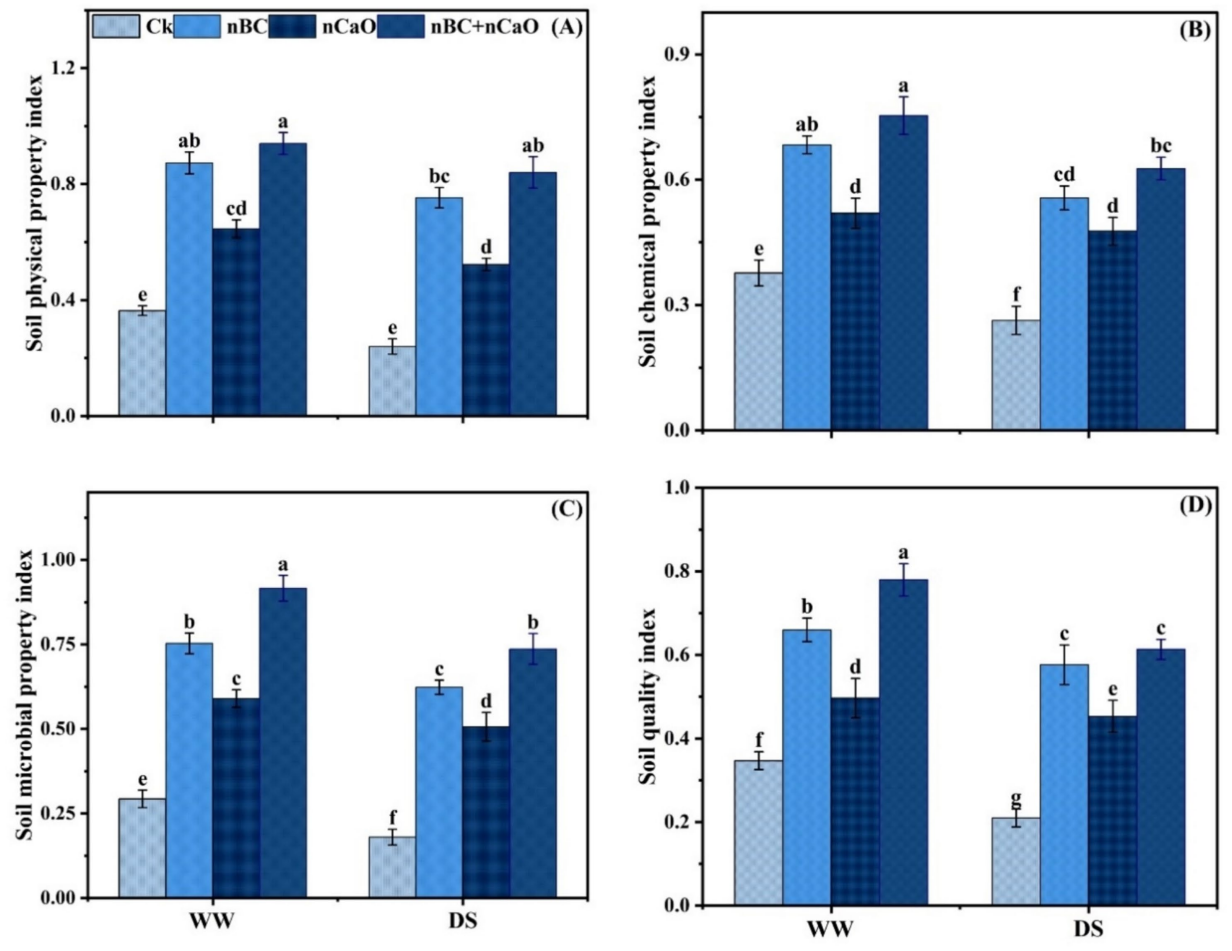
**(A)** Soil physical property index, **(B)** soil chemical property index, **(C)** soil biological property index, **(D)** soil quality index under synergistic application of nano biochar (nBC) and nano calcium oxide (nCaO) and drought treatments [well-watered (WW) and drought stress (DS)]. The values are mean ± SE of three replicates. Different lower-case letters indicate significant (*p* < 0.05) differences among treatments for WW and DS. Ck, control without nBC and nCaO.

Soil physical-, chemical-, biological- and quality-index were decreased under drought stress, compared with well-watered. However, nBC and nCaO significantly increased these indices under both well-watered and drought stress conditions. Notably, the combined nBC and nCaO significantly increased the soil physical index by 250.4%, chemical index by 137.9%, biological index by 309.1% and quality index by 192% compared with the respective control under drought stress (Figure 3).

### 3.3 Soil enzymatic activities

Soil enzymatic activities like urease, acid-phosphatase, nitrate reductase, β-glucosidase, catalase, phosphomonoesterase and *N*-acetyl-β-d-glucosaminidase were all reduced by drought stress. The quantitative concentrations of urease, acid-phosphatase, nitrate reductase, β-glucosidase, catalase, phosphomonoesterase and *N*-acetyl-β-d-glucosaminidase ranged from 10.35 to 16.80 mg kg^−1^, 65.17 to 82.00 mg kg^−1^, 3.91 to 8.83 mg kg^−1^, 1.10 to 1.66 mg kg^−1^, 0.21 to 1.57 mg kg^−1^, 3.00 to 5.23 mg kg^−1^, and 2.00 to 3.20 nmol g^−1^ min^−1^, respectively, across all treatments (Table 1). The combined nBC and nCaO achieved the highest increase in enzymatic activities recording 48.32, 13.34, 100.00, 43.37, 612.50, 61.30 and 43.65% increase in urease activity, acid-phosphatase, nitrate reductase, β-glucosidase, catalase, phosphomonoesterase and *N*-acetyl-β-d-glucosaminidase, respectively, compared with Ck, under drought stress. Interestingly, under both well-watered and drought stress conditions, sole BC application reduced the activities of acid-phosphatase and catalase relative to Ck.

**Table 1:** Effect of soil amendments with nano-biochar (nBC) and nano-calcium oxide (nCaO) on soil enzymatic activities under drought stress.

Drought treatments	Nano-amendments	Urease (nmol g^−1^ min^−1^)	Acid-P (nmol g^−1^ min^−1^)	Nitrate-R (nmol g^−1^ min^−1^)	β-glucosidase (nmol g^−1^ min^−1^)	Catalase (nmol g^−1^ min^−1^)	Phosphomonoesterase (nmol g^−1^ min^−1^)	NAG (nmol g^−1^ min^−1^)
Well-watered	Ck	4.53 ± 0.06b	2.30 ± 0.03b	1.72 ± 0.04c	0.039 ± 0.001c	804 ± 30c	0.41 ± 0.01c	2.45 ± 0.04d
	nBC	5.26 ± 0.11b	2.11 ± 0.01c	2.22 ± 0.07c	0.039 ± 0.00023c	744 ± 30c	0.50 ± 0.01b	2.50 ± 0.03c
	nCaO	6.16 ± 0.12a	2.44 ± 0.02ab	2.88 ± 0.07b	0.046 ± 0.0003b	3,899 ± 30b	0.55 ± 0.01b	2.91 ± 0.03b
	nBC + nCaO	6.66 ± 0.11a	2.59 ± 0.03a	3.50 ± 0.12a	0.052 ± 0.0003a	4,672 ± 30a	0.63 ± 0.01a	3.32 ± 0.03a
Drought stress	Ck	4.10 ± 0.12d	2.19 ± 0.03c	1.55 ± 0.08b	0.035 ± 0.001d	635 ± 30c	0.36 ± 0.01d	2.20 ± 0.05d
	nBC	4.85 ± 0.06c	2.06 ± 0.01d	2.01 ± 0.08b	0.037 ± 0.0003c	536 ± 0c	0.43 ± 0.01c	2.35 ± 0.03c
	nCaO	5.69 ± 0.08b	2.34 ± 0.02b	2.62 ± 0.07a	0.043 ± 0.00023b	3,720 ± 30b	0.49 ± 0.0.02b	2.69 ± 0.03b
	nBC + nCaO	6.09 ± 0.08a	2.48 ± 0.02a	3.11 ± 0.11a	0.050 ± 0.001a	4,524 ± 60a	0.58 ± 0.02a	3.17 ± 0.04a

Analysis of variance	Urease	Acid-P	Nitrate-R	*β*-glucosidase	Catalase	Phosphomon.	NAG
Drought (DS)	**	***	**	**	**	**	**
Nano amendments (NA)	**	**	*	**	**	**	**
DS × NA	ns	ns	ns	ns	ns	ns	ns

Means within a column followed by the same letter(s) are not significantly different (ns) for specific drought treatment (*p* > 0.05). The values are means ± standard errors of three replicates (*n* = 3). *p*-value indicates significance level based on two-way ANOVA. *P < 0.05, **p < 0.01, ***p < 0.001. Ck, control without nBC and nCaO. Acid-P, acid phosphatase; NAG, *N*-acetyl-β-d-glucosaminidase.

### 3.4 Soil ecosystem multifunctionality

**Figure 4:**
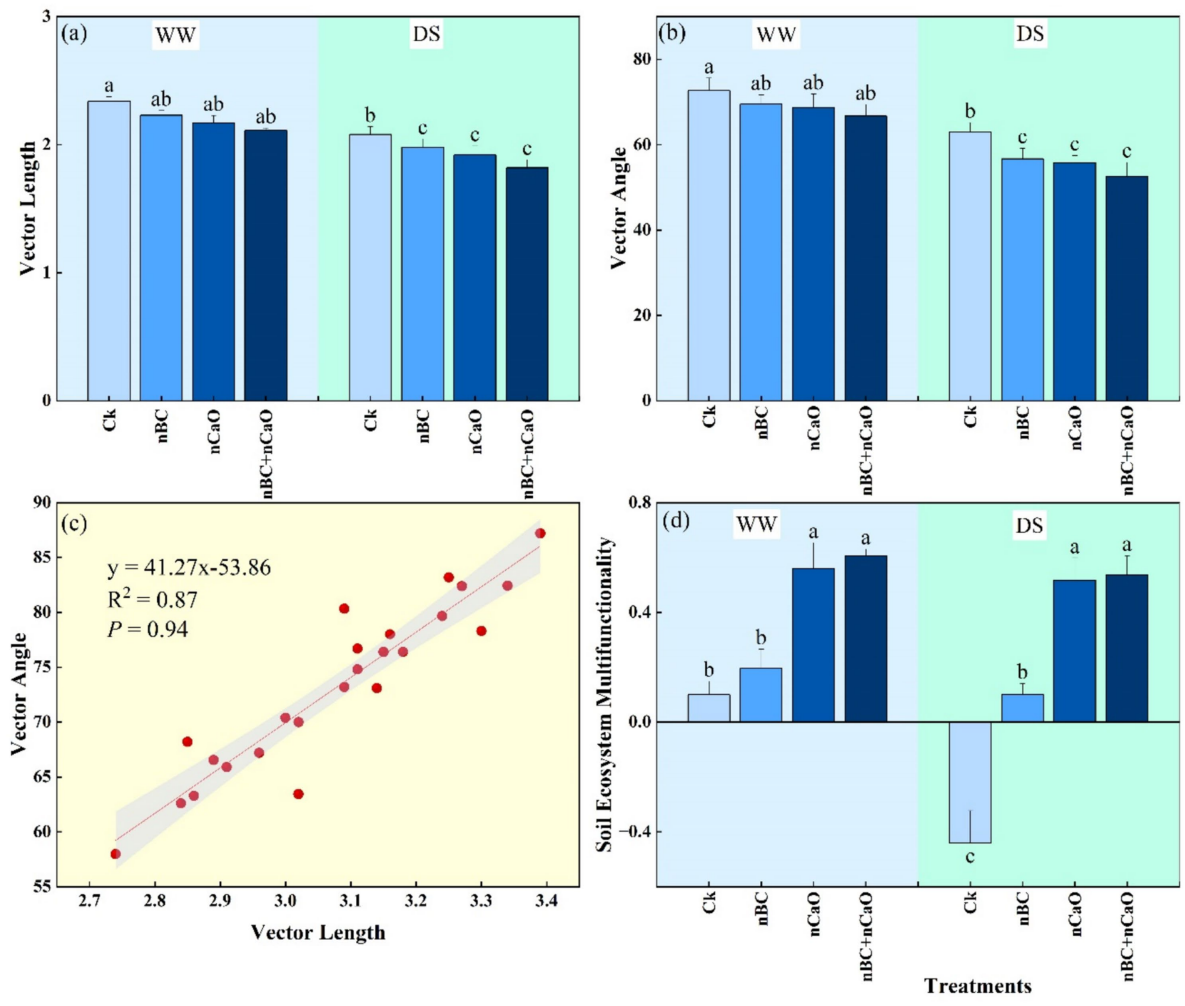
**(a)** Vector length, **(b)** vector angle, **(c)** relationship between vector angle and length, and **(d)** soil ecosystem multifunctionality under sole and co-application of nano biochar (nBC) and nano calcium oxide (nCaO), and drought treatments [well-watered (WW) and drought stress (DS)]. The values are mean ± SE of three replicates. Different lowercase letters indicate significant (*p* < 0.05) differences among treatments for WW and DS. Ck, control without nBC and nCaO.

Drought stress significantly reduced vector length, vector angle, and soil ecosystem multifunctionality (SEM) over well-watered conditions (Figure 4). The impact of nBC-nCaO amendments on vector angle and length was not significant. However, these amendments significantly increased SEM compared with respective control under both well-watered and drought stress conditions. The relationship between vector angle and length demonstrated *p*-values = 0.94 and *R*^2^ value = 0.87. The relationship between vector angle and length was linear showing positive effects of nBC and nCaO amendment under drought stress.

### 3.5 Alpha diversity

Microbial alpha diversity of archaea and bacteria was influenced by drought and nBC-nCaO treatments except for Shannon index under nBC-nCaO treatment. The interactive effect of these treatments was non-significant for all traits except for richness of archaea and Chao index in bacteria (Table 2). Drought stress significantly decreased the richness, Shannon and Chao index of both archaea and bacteria, compared to well-watered condition. However, nBC-nCaO treatments significantly increased these values under well-watered and drought stress conditions. Notably, combined nBC and nCaO increased archaea richness, Shannon and Chao index by 29.85, 24.54 and 106.60%, and bacterial richness, Shannon and Chao index by 19.24, 26.70 and 44.92%, respectively, under drought stress, compared with respective control without amendments (Table 2). Under both well-watered and drought stress conditions, overall alpha diversity indices followed the order: Ck > nCaO > nBC > nBC + nCaO.

**Table 2:** Effect of soil amendments with nano-biochar (nBC) and nano-calcium oxide (nCaO) on archaeal and bacterial abundance under drought stress.

Drought treatments	Nano-amendments	Archaea			Bacteria		
		Richness	Shannon	Chao	Richness	Shannon	Chao
Well-watered	Ck	141.14 ± 1.31d	2.76 ± 0.01d	342 ± 4.04d	2,803 ± 27.92d	7.28 ± 0.10b	3,534 ± 42.01d
	nBC	175.66 ± 1.80b	2.82 ± 0.01c	392.2 ± 3.56c	3,117 ± 35.37c	7.46 ± 0.05ab	4,439 ± 36.23c
	nCaO	187.03 ± 1.35a	3.14 ± 0.03b	492 ± 5.11b	3,186 ± 37.35b	7.54 ± 0.03ab	4,680 ± 31.85b
	nBC + nCaO	172 ± 1.96c	3.29 ± 0.02a	689.3 ± 10.6a	3,271 ± 29.87a	8.06 ± 0.12a	4,887 ± 35.24a
Drought stress	Ck	114 ± 1.66d	2.56 ± 0.10b	300 ± 7.63d	2,441 ± 23.67d	5.16 ± 0.10b	3,002 ± 34.70d
	nBC	153.8 ± 0.93b	2.70 ± 0.05ab	346.6 ± 5.08c	2,741 ± 36.39c	5.43 ± 0.06b	3,801 ± 29.16c
	nCaO	166.3 ± 1.05a	2.99 ± 0.08ab	444.7 ± 6.13b	2,796 ± 25.43b	5.69 ± 0.06b	4,103 ± 31.41b
	nBC + nCaO	148 ± 1.79c	3.19 ± 0.09a	620 ± 9.57a	2,910 ± 30.20a	6.54 ± 0.16a	4,351 ± 40.46a

Analysis of variance	Richness	Shannon	Chao	Richness	Shannon	Chao
Drought (DS)	***	**	**	**	**	**
Nano amendments (NA)	***	ns	**	**	**	**
DS × NA	**	ns	ns	ns	ns	**

Means within a column followed by the same letter(s) are not significantly different (ns) for specific drought treatment (*P* > 0.05). The values are means ± standard errors of three replicates (*n* = 3). *P*-value indicates significance level based on two-way ANOVA. **P < 0.01, ***P < 0.001. Ck, control without nBC and nCaO.

### 3.6 Nitrogen use efficiency and enzymatic activities

**Figure 5:**
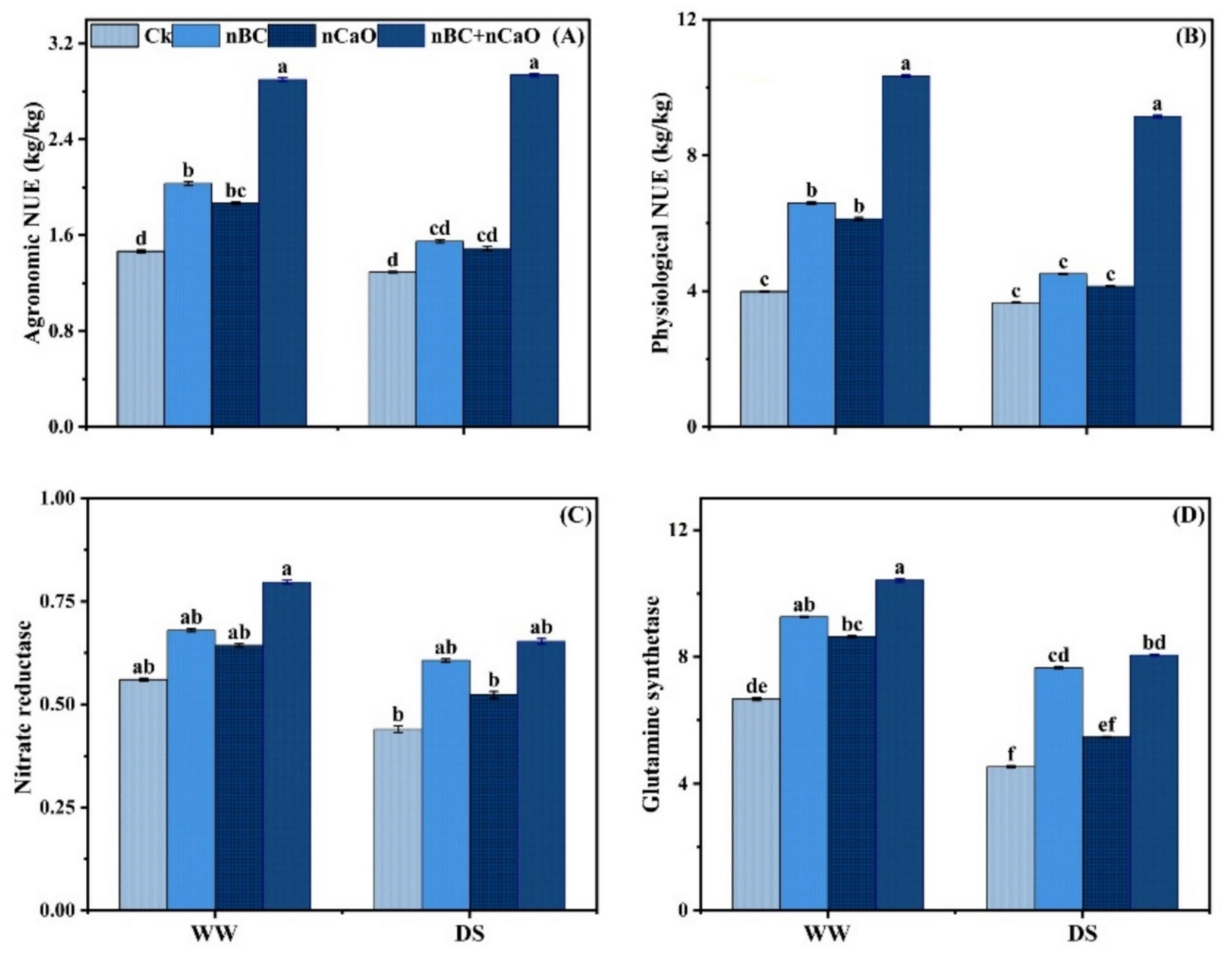
Impact of sole and co-applied nano biochar (nBC) and nano calcium oxide (nCaO) on **(A)** agronomic nitrogen use efficiency-NUE, **(B)** physiological NUE, **(C)** activity of nitrate reductase, and **(D)** glutamine synthetase under well-watered (WW) and drought stress (DS) conditions. The values are mean ± SE of three replicates. Different lower-case letters indicate significant (*p* < 0.05) differences among treatments for WW and DS. Ck, control without nBC and nCaO.

Agronomic and physiological nitrogen use efficiencies and nitrate reductase and glutamine synthetase activities were all decreased by drought stress (Figure 5). nBC and nCaO treatments significantly increased agronomic and physiological nitrogen use efficiencies compared to Ck. Notably, combined nBC and nCaO treatment caused most significant increase in agronomic and physiological nitrogen use efficiencies by 127.06 and 149.90%, respectively, in comparison to the Ck. Similarly, nitrate reductase and glutamine synthetase activities were significantly increased under combined nBC and nCaO amendment (Figure 5), with an increase of 48.48% in nitrate reductase, and 77.85% in glutamine synthetase, compared to Ck.

### 3.7 Relative gene expression

**Figure 6:**
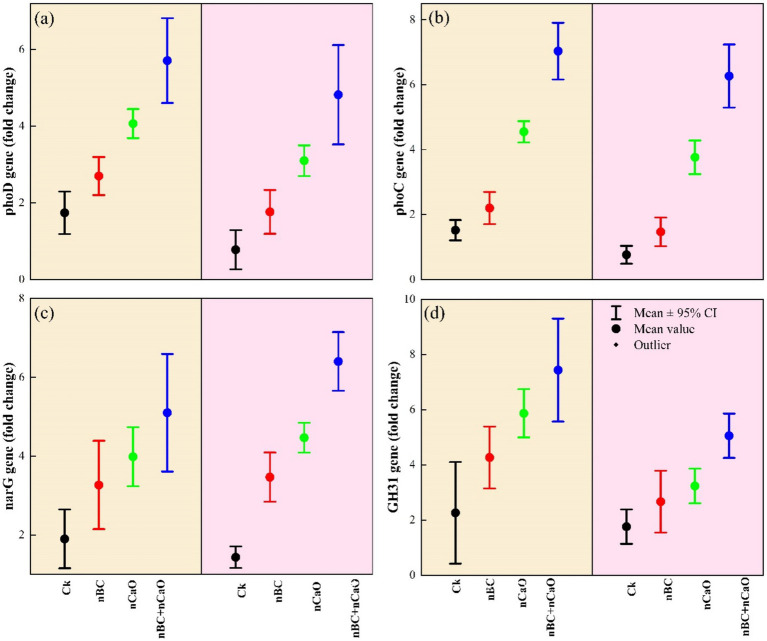
Impact of nano biochar (nBC) and nano calcium oxide (nCaO) as soil amendment on the expression of nitrogen, phosphorus, and carbon cycling genes (*phoD*, *phoC*, *narG*, and *GH31*) under well-watered (WW) and drought stress (DS) conditions **(a-d)**. Error bars on the graph indicate the standard error of the means (*n* = 3).

Drought stress and nBC-nCaO amendments significantly affected the expression of N-P-C cycling genes. The relative expression of these genes was relative less under drought stress than well-watered conditions (Figure 6). Combined nBC-nCaO amendments significantly increased the expression of *phoD*, *phoC*, *narG* and *GH31* genes under both well-watered and drought stress conditions. Notably, combined nBC and nCaO amendment demonstrated higher expression of these genes, showing 5.2-fold, 7-fold, 3.4-fold, and 1.8-fold increase in *phoD*, *phoC*, *narG* and *GH31*, respectively, compared with Ck, under drought stress. Under both well-watered and drought stress conditions, overall gene expression fold change followed the order: Ck > nCaO > nBC > nBC + nCaO.

### 3.8 Quality and yield traits

Tea quality traits, including polyphenols and total catechin contents, were decreased by drought stress. In addition, the impact of drought treatments was non-significant for caffeine and amino acid contents. Nonetheless, nutrient amendments significantly increased quality traits under well-watered and drought stress conditions. The quantitative concentrations of caffeine, polyphenol, total catechin and amino acid ranged from 3.67–4.35%, 182.30–250.30 mg kg^−1^, 8.66–11.00%, and 3.87–4.63%, respectively, across all treatments (Table 3). The combined nBC and nCaO achieved the highest improvement in quality traits recording 5.89, 12.24, 11.00 and 16.10% increase in caffeine, polyphenol, total catechin and amino acid content, respectively, compared with Ck, under drought stress. Similarly, the individual and interactive effects of drought and nutrient amendments were significant for all quality and yield traits, except for caffeine and amino acid contents. Drought stress significantly hampered plant height, leaf area and 10-bud weight; however, nBC-nCaO treatments improved these traits significantly under both well-watered and drought stress conditions (Table 3). Notably, combined nBC and nCaO significantly increased plant height, leaf area and 10-bud weight, recording 10.43, 97.55 and 42.53%, respectively, higher values than Ck, under drought stress (Table 3).

**Table 3:** Effect of soil amendments with nano-biochar (nBC) and nano-calcium oxide (nCaO) on tea quality and yield traits under drought stress.

Drought treatments	Nano-amendments	Caffeine (%)	Polyphenols (mg/kg)	Total catechin (%)	Amino acids (%)	Plant height (cm)	Leaf area	10-bud weight (g)
Well-watered	Ck	3.91 ± 0.02ab	197.3 ± 0.48ce	9.38 ± 0.01bc	4.17 ± 0.01b	86.33 ± 0.24de	5.84 ± 0.05d	14.35 ± 0.12d
	nBC	4.03 ± 0.01ab	209.6 ± 0.80bc	9.81 ± 0.02 ac	4.24 ± 0.01b	92.17 ± 0.29c	9.43 ± 0.04c	19.01 ± 0.13c
	nCaO	4.07 ± 0.01ab	223.0 ± 0.59b	10.17 ± 0.03ab	4.30 ± 0.02b	96.10 ± 0.26b	11.03 ± 0.03b	25.04 ± 0.04b
	nBC + nCaO	4.35 ± 0.01a	250.3 ± 0.20a	11.00 ± 0.03a	4.63 ± 0.03a	110.3 ± 0.39a	15.35 ± 0.07a	29.30 ± 0.11a
Drought stress	Ck	3.67 ± 0.01b	182.3 ± 0.45e	8.66 ± 0.03c	3.87 ± 0.01b	79.68 ± 0.33 g	3.00 ± 0.01e	11.96 ± 0.08e
	nBC	3.78 ± 0.01b	190.0 ± 0.80de	8.90 ± 0.03bc	4.32 ± 0.00a	82.0 ± 0.31f	3.90 ± 0.05e	13.19 ± 0.05de
	nCaO	3.84 ± 0.01ab	195.0 ± 0.83ce	9.22 ± 0.01bc	4.37 ± 0.01a	85.1 ± 0.30e	5.16 ± 0.02d	14.11 ± 0.07de
	nBC + nCaO	3.89 ± 0.01ab	204.6 ± 0.76 cd	9.62 ± 0.02bc	4.50 ± 0.01a	88.0 ± 0.36d	5.93 ± 0.04d	17.05 ± 0.14c

Analysis of variance	Caffeine	Polyphenols	Total catechin	Amino acids	Plant height	Leaf area	10-bud weight
Drought (DS)	ns	***	**	ns	**	**	**
Nano amendments (NA)	**	**	**	*	**	**	**
DS × NA	ns	*	**	ns	**	**	**

Means within a column followed by the same letter(s) are not significantly different (ns) at *p* > 0.05. The values are means ± standard errors of three replicates (*n* = 3). *P*-value indicates significance level based on two-way ANOVA. *P < 0.05, **P < 0.01, ***P < 0.001. Ck, control without nBC and nCaO.

### 3.9 Relationship

**Figure 7:**
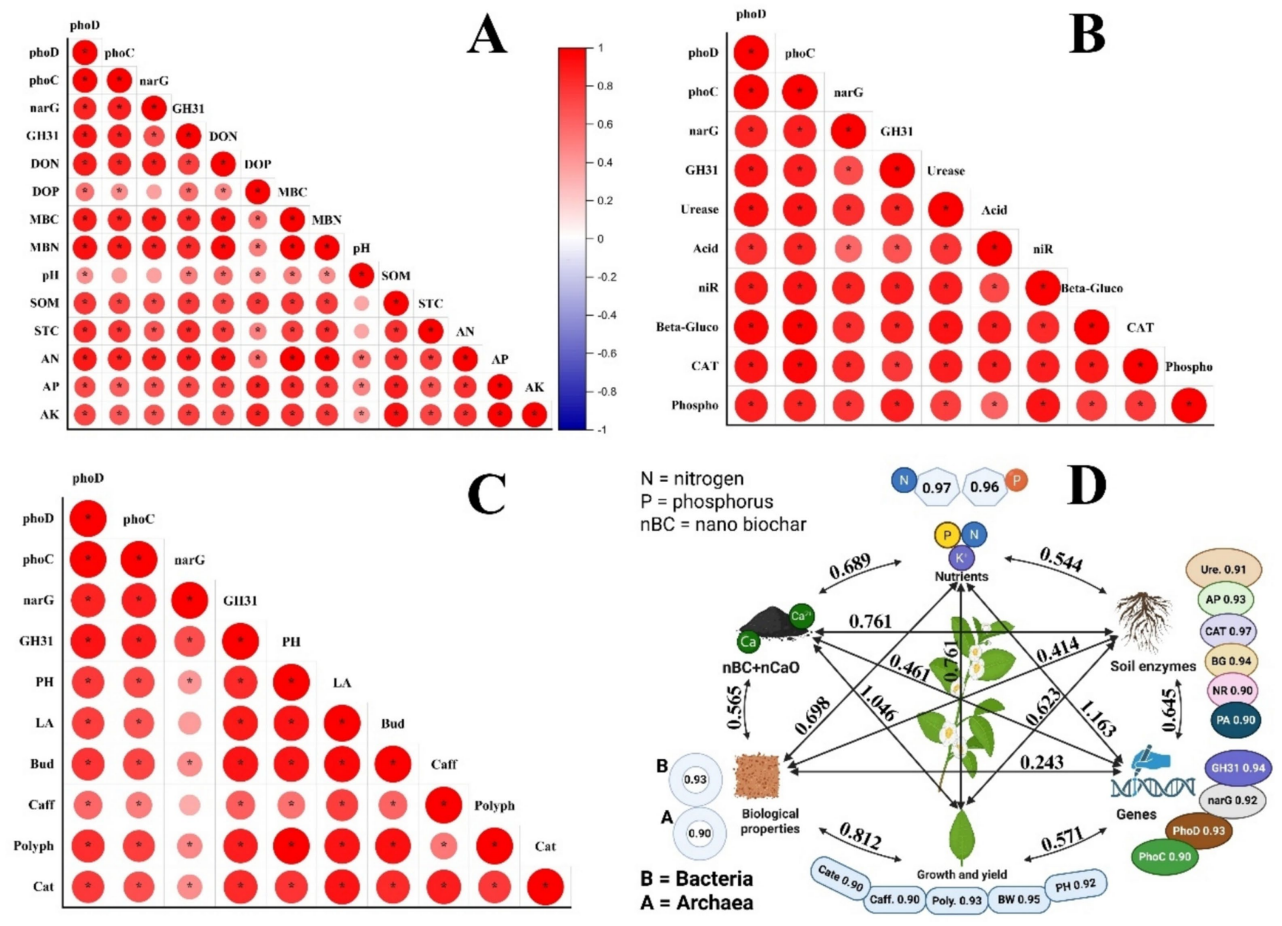
The relationship among gene expressions and **(A)** soil physico-chemical properties, **(B)** soil enzymatic activities, and **(C)** plant growth and quality traits under nano-biochar (nBC) and nano calcium oxide (nCaO) application under drought stress. **(D)** Structural equation modeling indicated both direct and indirect influence of simultaneous applied nBC and nCaO on soil nutrient uptake, soil enzymatic activities, biological properties, and gene expression and growth and quality traits of tea seedlings under drought stress. PH, plant height; LA, leaf area; Bud, bud weight; Caff, caffeine content; Poly, polyphenol content; Cate, catechins; Ure, urease; AP, acid phosphatase; NR, nitrate reductase; BG, β-glucosidase; CAT, catalase; PA, phosphomonoesterase.

In this study, the relationship between N-P-C cycling genes and soil physico-chemical and biological properties and enzymatic activities were studies (Figures 7A–C). All genes showed positive relationship with soil physico-chemical properties including DOP, DON, MBC, MBN, SOM, and STC. Moreover, soil available nutrients such as AN, AP and AK also demonstrated a strong positive association with N-C-P genes expression. However, there was a non-significant relationship between soil pH and expression of N-P-C cycling genes. Soil enzymatic activities demonstrated a strong positive association with the expression of these genes. Similarly, crop quality and yield related traits showed a positive correlation with all genes except for *narG*, which had no relationship with these traits.

Structural equational modeling demonstrated a strong background on the relationship among the recorded traits. For this study, we recorded a goodness-of-fit (GOF) value of 0.90 for drought stress (Figure 7D). Equational modeling showed that combined nBC and nCaO significantly and positively affected all recorded traits particularly soil enzymatic and microbial activities. Co-applied nBC and nCaO directly and positively affected nutrient availability (path coefficient = 0.689), soil enzyme activities (path coefficient = 0.761), biological properties (path coefficient = 0.565), gene expression (path coefficient = 0.623) and quality and yield traits (path coefficient = 1.040). Furthermore, biological properties demonstrated a substantial and positive effect on nutrient availability, and crop quality and yield. In addition, soil enzymatic activities depicted a strong and positive influence on biological properties, nutrient availability, gene expression and yield traits.

## 4 Discussion

Drought stress imposes severe constraints on tea (*Camellia sinensis* L. O. Kuntze) production by reducing soil moisture content, limiting nutrient availability, suppressing microbial activity, and impairing photosynthetic capacity, all of which reduce yield and quality (Moreno-Lora et al., 2023; Yuan et al., 2024). The findings of this study supported the hypothesis that the combined application of nBC and nCaO would act synergistically to stabilize soil physico-chemical properties and enhance key physiological pathways in tea plants, thereby improving nutrient acquisition and promoting growth under water-limited conditions.

In the current study, the combined application of nBC and nCaO markedly improved soil pH and increased concentrations of available N, P and K, alongside elevating soil enzyme activities and the abundance of archaeal population indicators. Mechanistically, the large surface area and abundant functional groups of nBC help buffer soil pH through surface complexation and proton exchange, while also adsorbing and gradually releasing mineral nutrients, thereby reducing nutrient leaching under drought stress (Jiang et al., 2023; Brar et al., 2024). Mehran et al. (2025) similarly reported that BC enhances soil nutrient mineralization through efficient slow-release mechanisms, ultimately improving crop yield. In our study, significant increases in soil enzyme activities related to organic N and P mineralization, as well as elevated archaeal markers, suggest that nBC created microhabitats that protected and promoted drought-tolerant microbial communities, thereby improving the nutrient cycling and root nutrient uptake (Dimitriadou et al., 2025). Consistent with this, Lian et al. (2022) found that biochar amendments improve soil structure and create more favorable microbial niches, with nanoscale materials further enhancing these effects by increasing the reactive interface with the soil solution.

In the current study, we observed a significant increase in N use efficiency and NR synthesis under the combined nBC and nCaO treatment. This enhancement can be attributed to two primary mechanisms. First, nBC retained mineral N in plant-available forms, thereby maintaining a steady substrate supply for NR activity and amino acid biosynthesis, even under reduced soil moisture conditions where mass flow is limited (Mehmood et al., 2024; Ying et al., 2025). In addition, some studies have demonstrated that nCaO helps plant to activate calcium-dependent protein kinases and transcriptional networks when act as a signaling ion. These kinases and networks help elevate the expression levels of NR genes and stabilize correlated enzyme complexes (Michailidis et al., 2019; Mazhar et al., 2023). Furthermore, calcium-induced signaling expanded the efficiency with which available N was embraced into organic pools; this phenomenon is crucial for reducing N losses from the system and enhancing overall N use efficiency (Weng et al., 2022). Recently, Naz et al. (2024) demonstrated that calcium also acts as a secondary messenger, thus it moderates cytosolic Ca^2+^ transients that originate downstream phosphorylation cascades. The involvement of these cascades in elevating the synthesis and post-translational stability of N-assimilating enzymes has been well studied. Elevated synthesis of these enzymes ultimately helps in linking soil-mediated N conservation with intrinsic metabolic activation.

The results of this work clearly indicated that concurrent application of nBC and nCaO into the soil has improved catechin and total polyphenol levels in tea leaves. These improvements in catechin and polyphenol concentrations can be explained through increased carbon and N assimilation as well as decreased oxidative stress damage (Savchenko and Tikhonov, 2021; Gulsah et al., 2025). In addition, our results have also showed that nBC and nCaO synergies resulted in elevated levels of NR and photosynthetic pigments under water limited conditions. These improvements indicate that enhanced availability of substrate, particularly amino acids and carbohydrate precursors, along with stabilized redox homeostasis can lead to elevated metabolic capacity (Li et al., 2018; Peña Calzada et al., 2022).

This response can be explained through calcium-involved activation of enzymatic and non-enzymatic activities, which help sustain the accumulation of ROS under stressful environments. These sustainable activities further support the reduced oxidative degradation of biosynthetic enzymes. Previous studies have documented that these protections preserved metabolic flux associated with phenylpropanoid pathway; the involvement of these pathways in catechin and polyphenol biosynthesis has been well documented previously (Aslam et al., 2021; Ikram et al., 2025). Recently, Shahzadi et al. (2025) demonstrated that lower oxidative marker levels and preserved enzyme functionality, predominantly phenylalanine ammonia-lyase and other downstream enzymes, permit the constant translation of primary metabolites into secondary metabolites that harvest the overall seedling quality under numerous environmental stresses.

In this work, synergistic applied nBC and nCaO also depicted significantly heavier bud weight and increased plant height under drought stress. These improvements can be explained through elevated availability of soil N (Badr and Brüggemann, 2020). Furthermore, calcium-induced maintenance of thylakoid membranes and photosynthetic protein complexes results in better performance of light-harvesting systems from dehydration-induced disassembly under drought stress (Bilge et al., 2025). The upsurges in bud weight and increase in plant height were positively correlated with elevated water retention, resulting from improved soil porosity, better aggregate stability, and enhanced water-holding capacity under nBC addition (Shafiq et al., 2023; Shiade et al., 2024). Calcium-induced osmotic and structural modifications within leaf and bud tissues further supported these effects. It has been well demonstrated that nBC realistically maintains water and releases it gradually, thus supporting cellular performance and upholding phloem transport of produced assimilates to emerging buds (Mehmood et al., 2024). These processes are crucial to promote cell wall cross-linking and membrane stability. Subsequently, this phenomenon facilitates cell expansion and provides mechanical reinforce under abiotic stresses (Gulsah et al., 2025). In crux, these Ca-induced mechanisms uphold photosynthetic carbon gain and confirm its distribution to apical and reproductive sinks. Increased distribution has resulted in increasing bud mass and enhancing shoot length under stress environment (Minghan et al., 2025).

Overall, the findings of this study demonstrated the concurrent effects of soil applied nBC and nCaO on soil and crop performance. The combined addition of these amendments supports N metabolism, antioxidative protection, and overall improvements in the agronomic traits and quality indices of tea under drought stress. Our results demonstrate that the synergistic applied nBC and nCaO tackles challenges such as drought-brought substrate constraints, disrupted signaling, and oxidative damage. Mechanistically, nBC and nCaO application elevated soil pH, the availability of rhizospheric N, P, and K. These improvements further enhance the activities of soil enzymes, archaeal abundance and leaf enzymatic activities including NR. A significant improvement in tea biochemical traits such as catechins, polyphenols, amino acid, and growth parameters has also been observed under drought stress. These amendments appear to act across complementary soil and plant processes, highlighting the significance of further investigating the dose–response relationships and optimum effectiveness of these amendments to maximize profits while monitoring long-term soil health.

## 5 Conclusion

The combined application of nBC and nCaO signifies an applicable and viable approach for lessening drought induced damage and improving soil health and crop performance. Their combined addition into the soil markedly increased soil pH and the availability of rhizospheric nutrients such as soil organic matter, total carbon, and available nitrogen. Concurrent application of nBC and nCaO also resulted in elevated soil enzyme activities and improved soil microbial functioning under drought stress condition. In addition, the combined soil incorporation of nBC and nCaO has upregulated the expression of N-P-C cycling genes as well as a significant improvement in tea quality and yield traits under drought stress. Overall, these results emphasize the potential of combined application of nBC and nCaO in alleviating drought stress and enhancing tea productivity. However, further long-term field experiments are needed to support the practical applicability of these organic nutrient amendments in drought-prone areas.

## Data Availability

The original contributions presented in the study are included in the article/supplementary material, further inquiries can be directed to the corresponding authors.
